# *In situ* photodeposition of ultra-small palladium particles on TiO_2_

**DOI:** 10.1107/S1600577524004788

**Published:** 2024-07-15

**Authors:** Elizaveta Kozyr, Sara Martí-Sánchez, Alina Skorynina, Jordi Arbiol, Carlos Escudero, Lorenzo Mino, Aram Bugaev

**Affiliations:** a Department of Chemistry and NIS Centre University of Torino Via Giuria 7 10125Torino Italy; b Catalan Institute of Nanoscience and Nanotechnology CSIC and BIST, Campus UAB 08193Bellaterra Barcelona Spain; c ALBA Synchrotron Light Source Carrer de la Llum 2-26, Cerdanyola del Vallès 08290Barcelona Spain; d ICREA Pg. Lluís Companys 23 08010Barcelona Spain; e Paul Scherrer Institute Forschungstrasse 111 5232Villigen Switzerland; ESRF – The European Synchrotron, France

**Keywords:** photocatalysis, Pd/TiO_2_, *operando* spectroscopy, XANES

## Abstract

*In situ* and *operando* investigation of photocatalysts plays a fundamental role in understanding the processes of active phase formation and the mechanisms of catalytic reactions, which is crucial for the rational design of more efficient materials. Using a custom-made *operando* photocatalytic cell, an *in situ* procedure to follow the formation steps of Pd/TiO_2_ photocatalyst by synchrotron-based X-ray absorption spectroscopy (XAS) is proposed. The procedure resulted in the formation of ∼1 nm Pd particles with a much narrower size distribution and homogeneous spreading over TiO_2_ support compared with the samples generated in a conventional batch reactor. The combination of *in situ* XAS spectroscopy with high-angle annular dark-field scanning transmission electron microscopy demonstrated the formation of single-atom Pd(0) sites on TiO_2_ as the initial step of the photodeposition process. Palladium hydride particles were observed for all investigated samples upon exposure to formic acid solutions.

## Abstract 2

An *in situ* approach to generate photocatalysts using a custom-made photocatalytic cell allowed, by synchrotron-based X-ray absorption spectroscopy, the different stages of Pd nucleation onto TiO_2_ samples to be followed yielding a highly homogeneous distribution of 1 nm palladium nanoparticles.

## 1. Introduction

Carlo Lamberti, whether as a teacher, a supervisor or a colleague of us, had led numerous research initiatives that our team is still actively pursuing. Those mainly include studies of catalysts represented by supported metal nanoparticles (Bugaev *et al.*, 2013 ▸, 2016 ▸; Bugaev, Guda *et al.*, 2019 ▸) or functionalized metal-organic frameworks (Braglia, Borfecchia, Martini *et al.*, 2017 ▸; Braglia, Borfecchia, Lomachenko *et al.*, 2017 ▸; Bugaev, Skorynina *et al.*, 2019 ▸; Skorynina *et al.*, 2024 ▸; Pnevskaya & Bugaev, 2023 ▸; Pnevskaya *et al.*, 2021 ▸; Kamyshova *et al.*, 2020 ▸; Lomachenko *et al.*, 2018 ▸; Gallo *et al.*, 2017 ▸; Usoltsev *et al.*, 2024 ▸) under reaction conditions. Highlighting the role of *in situ* and *operando* spectroscopies for in depth understanding of functional materials, these approaches were successfully applied to real industrial catalysts (Fang *et al.*, 2021 ▸; Groppo *et al.*, 2018 ▸; Bugaev *et al.*, 2021 ▸), and were further extended to investigate high-pressure industrially relevant processes (Janssens *et al.*, 2022 ▸; Ramirez *et al.*, 2021 ▸). In many of the aforementioned works, the catalysts themselves were also synthesized *in situ*, followed by the application of the catalytic reaction conditions, and Carlo was one of the key actors on the synchrotron-world scene, advancing the development of *in situ* and *operando* methodologies. During the 2018 Faraday Discussion conference (Adishev *et al.*, 2018 ▸), he was suggesting pushing the development of a new direction, calling it ‘*construendo*’ or ‘*aedificando*’, analogous to *operando* spectroscopy, to focus on the formation steps of nanosystems during their synthesis. Although that proposal was made with a touch of Carlo’s playful and adventurous spirit, the topic was, and remains, of significant importance, supported by numerous successful examples in the literature.

Application of X-ray absorption spectroscopy (XAS), one of Carlo’s biggest passions (van Bokhoven & Lamberti, 2016 ▸; Mino *et al.*, 2013 ▸; Giannini *et al.*, 2020 ▸), in heterogeneous catalysis has long become a widely used and standard tool, despite the need of using synchrotron radiation to follow fast processes (Abdala *et al.*, 2012 ▸; Weckhuysen, 2003 ▸; Bugaev *et al.*, 2024 ▸), while for slow processes, *i.e.* hours or days, laboratory XAS can be applied (Sherborne & Nguyen, 2015 ▸). The area of photocatalysis, however, remains more demanding for several reasons, among which are (i) the typical presence of a liquid phase, (ii) lower concentrations of active metallic species with respect to the substrate, and (iii) a more complicated design of the *in situ*/*operando* cells (Piccolo *et al.*, 2020 ▸; Khare *et al.*, 2020 ▸; Issa Hamoud *et al.*, 2022 ▸). If the photocatalyst is suspended in a solution, the detection of XAS signal is complicated; therefore, an approach when the catalyst is deposited on a window of an *in situ* cell is more often exploited (Mu *et al.*, 2023 ▸; Haselmann *et al.*, 2020 ▸). However, due to the substantial difference in penetration depth between UV or visible light and hard X-ray photons, it is possible that only a minor fraction of the species probed by XAS are actually involved in the photocatalytic process, therefore making structure–activity correlations irrelevant (Jacobs *et al.*, 1989 ▸).

This contribution describes the *in situ* synthesis of a Pd/TiO_2_ catalyst in a home-made photocatalytic cell. The process of *in situ* photodeposition of Pd and the formation and evolution of different Pd species during synthesis are probed by synchrotron-based XAS under reaction conditions. Furthermore, the morphology of the *in situ* prepared catalyst and its counterpart, made in a conventional batch reactor with suspended TiO_2_, is investigated by high-angle annular dark-field scanning transmission electron microscopy (HAADF-STEM). The combination of *in situ* XAS spectroscopy with *ex situ* HAADF STEM has been demonstrated as a powerful workflow for detailed investigation of the formation mechanisms of the current catalysts (Pastor *et al.*, 2024 ▸).

## 2. Materials and methods

### 2.1. Chemicals

*Ex situ* Pd/TiO_2_ samples were prepared using P25-type TiO_2_ (Evonik Operations GmbH) as a support. Palladium atomic absorption standard solution (1 mg ml^−1^ Pd in 5% HCl, Sigma-Aldrich) was used as a metal source. Both the support and the metal source, in a ratio to obtain 2 wt% Pd/TiO_2_, were dissolved in 0.1 *M* solution of formic acid in Mili-Q water with a total volume of 100 ml inside a batch-type quartz reactor cell, which was permanently flushed with N_2_. After 15 min of flushing, the solution was irradiated by UV light (λ = 365 nm) for 20 min with irradiance *E* = 30 W m^−2^.

*In situ* Pd/TiO_2_ samples were prepared directly during the XAS experiment (see below). First, the P25 powder was dissolved in water and the resulting TiO_2_ ink (∼15 mg of dry powder, measured for each sample) was deposited on the window of the home-made *operando* photocatalytic cell (Kozyr *et al.*, 2023 ▸) and left drying. The cell was then filled with a mixture of 15 ml of Mili-Q water and 10 µL of formic acid. The amount of Pd source (1 mg ml^−1^ Pd in 5% HCl, Sigma-Aldrich) was calculated for every sample (∼300 µL) depending on the amount of TiO_2_, in order to reach the theoretical maximal loading of 2 wt% Pd/TiO_2_. The cell was tightly sealed and permanently flushed with He at 20 ml min^−1^, the outlet being monitored by a Cirrus 3-XD mass spectrometer (MKS Instruments). Then, the cell window was irradiated by a remotely controlled UV LED source (λ = 365 nm). After 20 min of total exposure to UV radiation, an additional 1 ml of formic acid was added to the cell as a hole scavenger and the cell was continuously exposed to UV irradiation.

### 2.2. *In situ* XAS measurements

*In situ* Pd *K*-edge XAS data were collected at the NOTOS beamline of ALBA synchrotron (Spain). The NOTOS beamline source is a 1.42 T bending magnet with 8.5 keV critical energy and a maximum flux of 10^11^ photons s^−1^ at this critical energy. The beamline optics consists of a cylindrical mirror (M1) with downward orientation, with a Si and a Rh stripe, that collimates the beam onto a double-crystal monochromator (DCM) made by CINEL. The DCM is a direct drive monochromator with fixed exit and two pairs of crystals, Si(111) and Si (220), enabling an energy range between 4.7 and 30 keV. The beam coming from the DCM impinges on an Rh-coated double-channel toroidal mirror (M2, with 34 and 55 mm sagittal radii of curvature), which allows for horizontal focusing of the beam on the sample.

**Figure 1:**
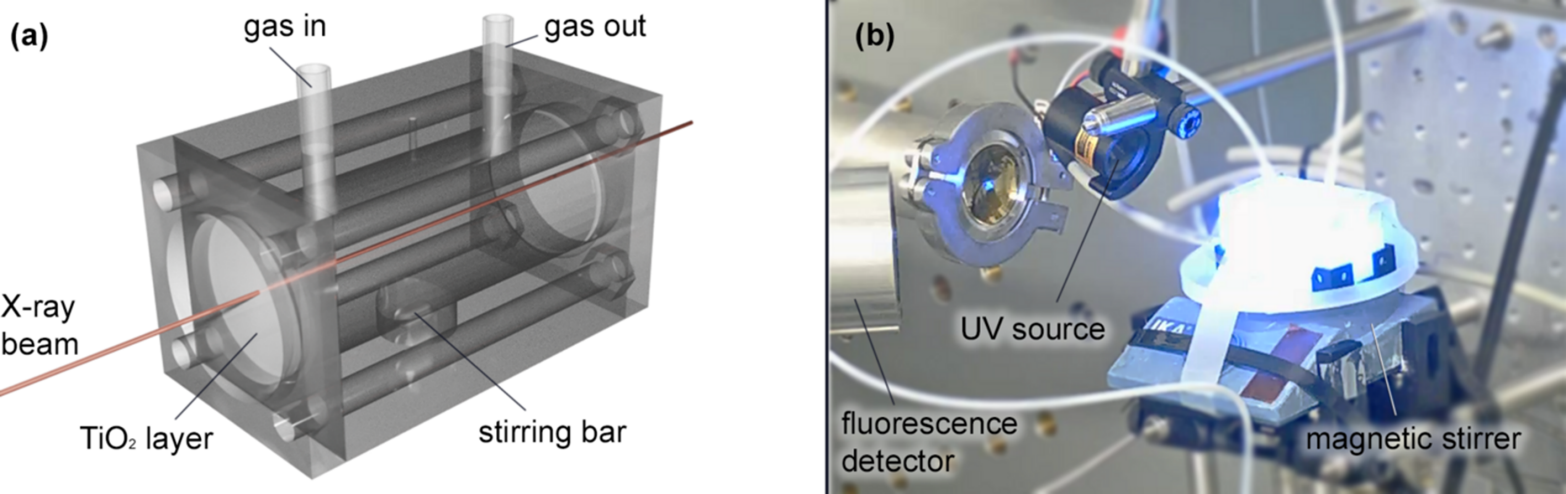
Schematic (*a*) and photograph (*b*) of the *operando* photocatalytic cell.

For this particular experiment, the beamline optics was optimized for the Pd *K*-edge (24.35 keV) by using the Rh-coated stripe of M1, the Si(111) crystals pair and the 34 mm channel of M2. The beam size dimensions were 250 µm × 370 µm (H × V) approximately. The DCM was operated in continuous scanning mode and the energy was scanned within the 24.2–24.9 keV range (∼1 min per spectrum). The cell window was oriented perpendicularly to the incoming X-ray beam, allowing the collection of both fluorescence and transmission signals. The transmission measurements were acquired with three ion chambers as detectors with a proper gas mixture, optimized to improve the measured signal (absorbing ∼15%, 85% and 85%, respectively). The home-made cell was placed between the first and second ionization chambers, and a Pd metallic reference foil was placed between the second and the third to check for energy calibration. The fluorescence detector, a 13-channel Si drift detector from Mirion Technologies (Canberra Olen), was oriented at ∼45° with respect to the incoming beam, from the same side as the UV source (see Fig. 1 ▸).

Among different cell windows materials, cellulose acetate film demonstrated the highest stability under the beam, while for other types of windows (quartz glass, Mylar film) beam-induced implantation of palladium into window material was observed during beam damage tests. To further reduce the beam effects, the cell was automatically moved by 0.5 mm in the horizontal direction (and when the row is finished, by 0.5 mm vertically) after each XAS spectrum. This procedure limited the maximum exposure of any fraction of the sample to the X-ray beam to ∼1 min and showed that no palladium nanoparticles were formed on the window in the absence of UV light. XAS data processing was carried out using the *Demeter* package (Ravel & Newville, 2005 ▸).

### 2.3. HAADF-STEM measurements

HAADF-STEM micrographs and energy-dispersive X-ray spectroscopy (EDX) elemental maps were acquired in a double-corrected Spectra 300 STEM microscope (ThermoFisher Scientific) operated at 300 kV. Histograms of particle size distribution were obtained by measuring 150–250 particles for each sample. For sample deposition, powder samples were dispersed in water, sonicated and deposited on lacey carbon Cu grids with an ultrathin carbon layer.

## 3. Results and discussion

### 3.1. *In situ* photodeposition of Pd on TiO_2_

Two general strategies can be exploited when performing photocatalytic reactions (Wei *et al.*, 2022 ▸). In the first one, the photocatalyst is dispersed or suspended in a solution, which is believed to result in a better mass transfer. However, the distribution of light from the external source through the volume of such reactors is typically inhomogeneous. An alternative approach uses an immobilized catalyst, typically deposited on a UV-transparent window, which can be easily illuminated in a constant manner. The latter is also preferable for XAS studies, as the amounts of catalyst and/or the cell dimensions are typically incompatible with data collection in transmission geometry, while deposition of the catalyst on the window of a photocatalytic cell allows for fluorescence data collection. Still, another problem remains due to the fact that the penetration depth of X-rays within TiO_2_ varies from a tenth or hundreds of micrometres at *K*-edges of 3*d* elements or *L*_3_-edges of 5*d* elements to millimetres for 4*d* elements, while the penetration depth of UV light into TiO_2_ is typically in the nanometre to micrometre range. As a result, the majority of metal species, that are easily probed by the X-ray beam, may not be accessible by UV radiation and remain inactive for the photocatalytic reactions. This issue can be solved by precise control of the thickness of the photocatalyst or by *in situ* photodeposition of metals on TiO_2_.

Here, we focus on the second approach, in which Pd was *in situ* photodeposited on TiO_2_. The procedure was performed using a home-made *operando* cell shown in Fig. 1 ▸. A thin layer of TiO_2_ was first applied on the front cell window and the cell was then filled with a solution of Pd-precursor under continuous stirring and flushing with He. The UV radiation was applied from the outside of the cell to the front window by setting the irradiance to 400 W m^−2^. The photodeposition of Pd onto the TiO_2_ layer was probed by XAS in fluorescence mode from the same side. The advantages of this approach are: (i) the homogeneous irradiance of the catalyst over time; (ii) the distribution of the Pd species only in the UV-accessible part of TiO_2_, *i.e.* all Pd/TiO_2_ species can be further considered to be photoactive; and (iii) the minimization of the possible direct interaction of UV light with Pd species in solution.

**Figure 2:**
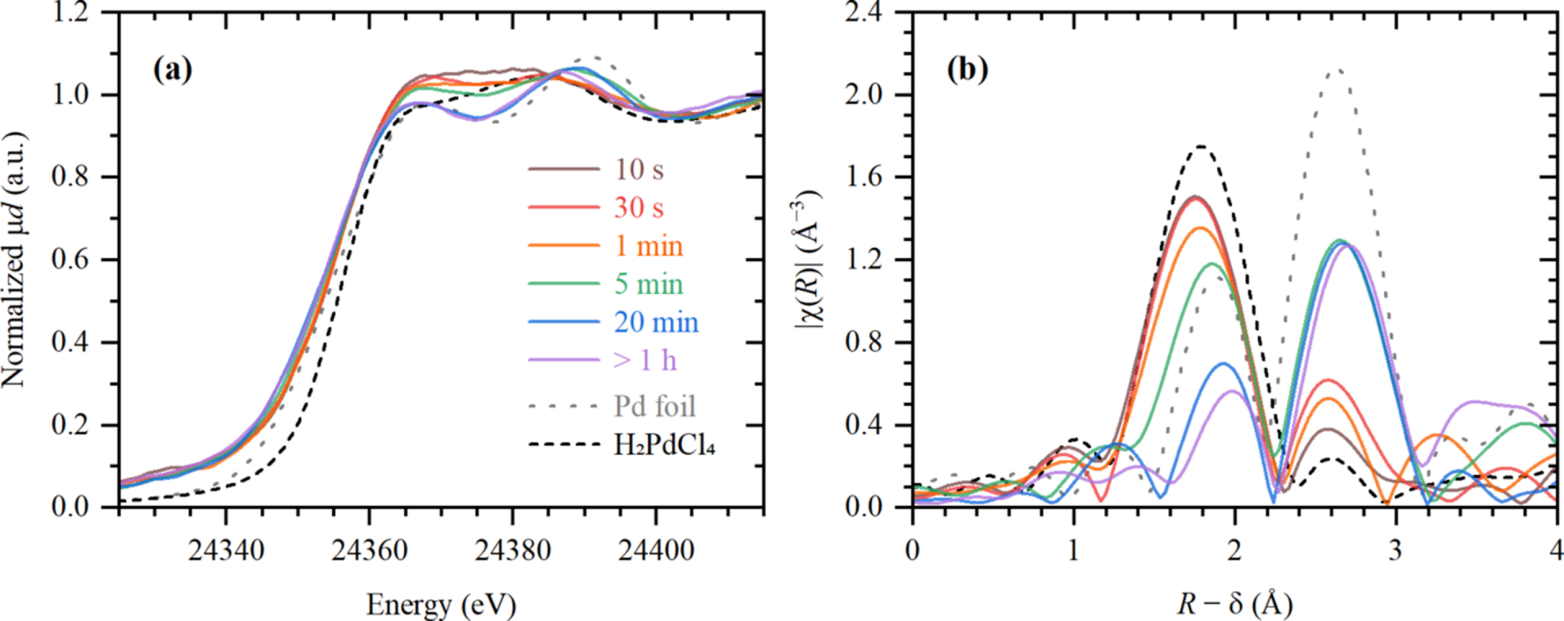
XANES (*a*) and phase-uncorrected FT-EXAFS (*b*) data collected during *in situ* photodeposition of palladium on TiO_2_. Colored lines correspond to different irradiation times. Dashed black and gray lines correspond to the reference H_2_PdCl_4_ and Pd foil, respectively.

To follow the progressive growth and evolution of Pd species on TiO_2_ upon photodeposition, the UV irradiation was switched on for 10 s, 20 s, 30 s, 4 min and 15 min, resulting in cumulative irradiation times of 10 s, 30 s, 1 min, 5 min and 20 min, respectively. After each step, XAS spectra were collected to probe the state of the photodeposited Pd. Fig. 2 ▸ shows the evolution of XANES and EXAFS data indicating a gradual formation of metallic Pd species, as compared with the reference Pd foil. Indeed, the photodeposition process was accompanied by the progressive formation of distinct XANES features, characteristic of metallic Pd, a decrease of the first-shell Pd–Cl contribution in EXAFS and a growth of the Pd–Pd one. Due to the nanometric dimensions, the intensity of the latter was smaller with respect to the Pd reference foil. The average Pd–Pd coordination number obtained through EXAFS fitting was 7.9 ± 0.8, corresponding to an average particle size of ∼1.2 nm, assuming their face-centred-cubic-like structure.

At the same time, a decrease of the edge jump at the Pd*K*-edge in the transmission signal was observed. This was explained by the fact that the transmission signal was initially dominated by the Pd species homogeneously distributed in the solution; however, when Pd was being deposited on the TiO_2_ layer, which was inhomogeneous both in its thickness and distribution over the window, this inhomogeneity led to a lower edge jump. The trend estimated based on this fact is shown in Fig. S1 of the supporting information while the results of elemental composition analysis from EDX data are reported in Table S1.

Interestingly, already after 10 s the edge position in XANES corresponded to the Pd(0) oxidation state [Fig. 2 ▸(*a*)]. Moreover, this spectrum could not be reproduced by a linear combination of the reference spectra of H_2_PdCl_4_ (the spectrum of this reference was identical to that of the precursor as shown in Fig. S2) and Pd foil, which is shown by the absence of isosbestic points in these spectra. This gives an important insight into the formation mechanism of Pd/TiO_2_ catalysts, suggesting that the photoreduction of Pd started with formation of isolated Pd(0) species, followed by a subsequent growth of Pd nanoparticles, most likely around those initial Pd(0) sites. The signals of H_2_ and CO_2_, due to the decomposition of formic acid, was observed in mass spectrometry data (Fig. S3) during each UV illumination.

Finally, we observed that the average Pd–Pd interatomic distance in the formed nanoparticles was 2.81 ± 0.01 Å, which is significantly higher than the value of 2.74 ± 0.01 Å determined for Pd foil. The shaping of XANES spectra, namely the positions and relative intensities of the first two maxima, indicated that such elongation was caused by the formation of palladium hydride (Bugaev *et al.*, 2013 ▸, 2016 ▸), while the presence of a carbide like phase was unlikely (Bugaev, Guda *et al.*, 2019 ▸; Usoltsev *et al.*, 2024 ▸).

**Figure 3:**
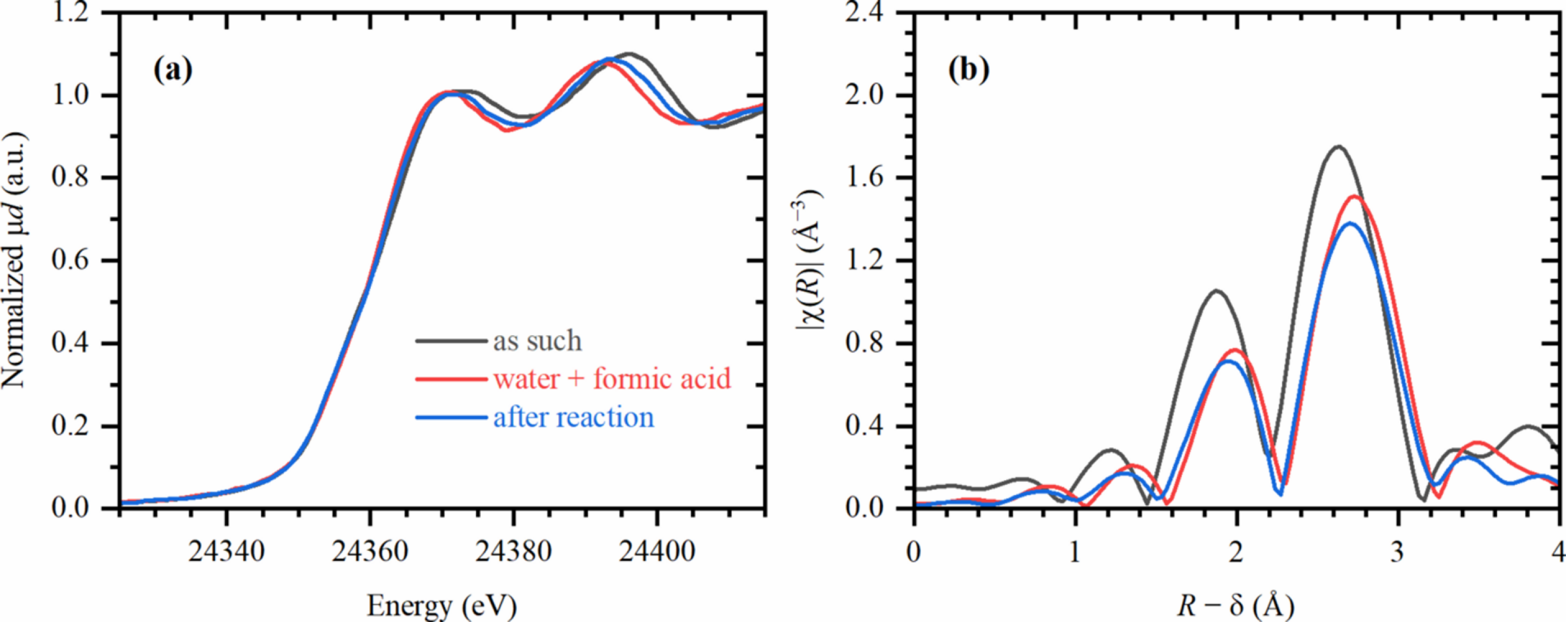
XANES (*a*) and phase-uncorrected FT-EXAFS (*b*) data for the sample, synthesized using a conventional batch procedure, in the initial state (black), after addition of water and formic acid to the photocatalytic cell (red) and after performing UV irradiation and removing the liquid (blue).

The sample prepared by conventional batch synthesis was also investigated and was applied to the window of the photocatalytic cell in a similar way, but already as Pd/TiO_2_. The average Pd–Pd coordination number for this sample was higher (11.6 ± 0.6) indicating that bigger particles were present. Upon the addition of the formic acid solution, the typical XANES features of palladium hydride also appeared [Fig. 3 ▸(*a*)] and the average Pd–Pd interatomic distances increased from 2.73 ± 0.01 Å to 2.84 ± 0.01 Å, which is visually shown in Fig. 3 ▸(*b*). The fact that the elongation of the interatomic distance upon formation of the palladium hydride phase was larger for this sample, compared with the one generated *in situ*, correlates with the assumption of a larger particle size. Interestingly, the hydride features in XANES and increased Pd–Pd distances remained even after the solution was removed.

### 3.2. Morphology of Pd/TiO_2_ samples

**Figure 4:**
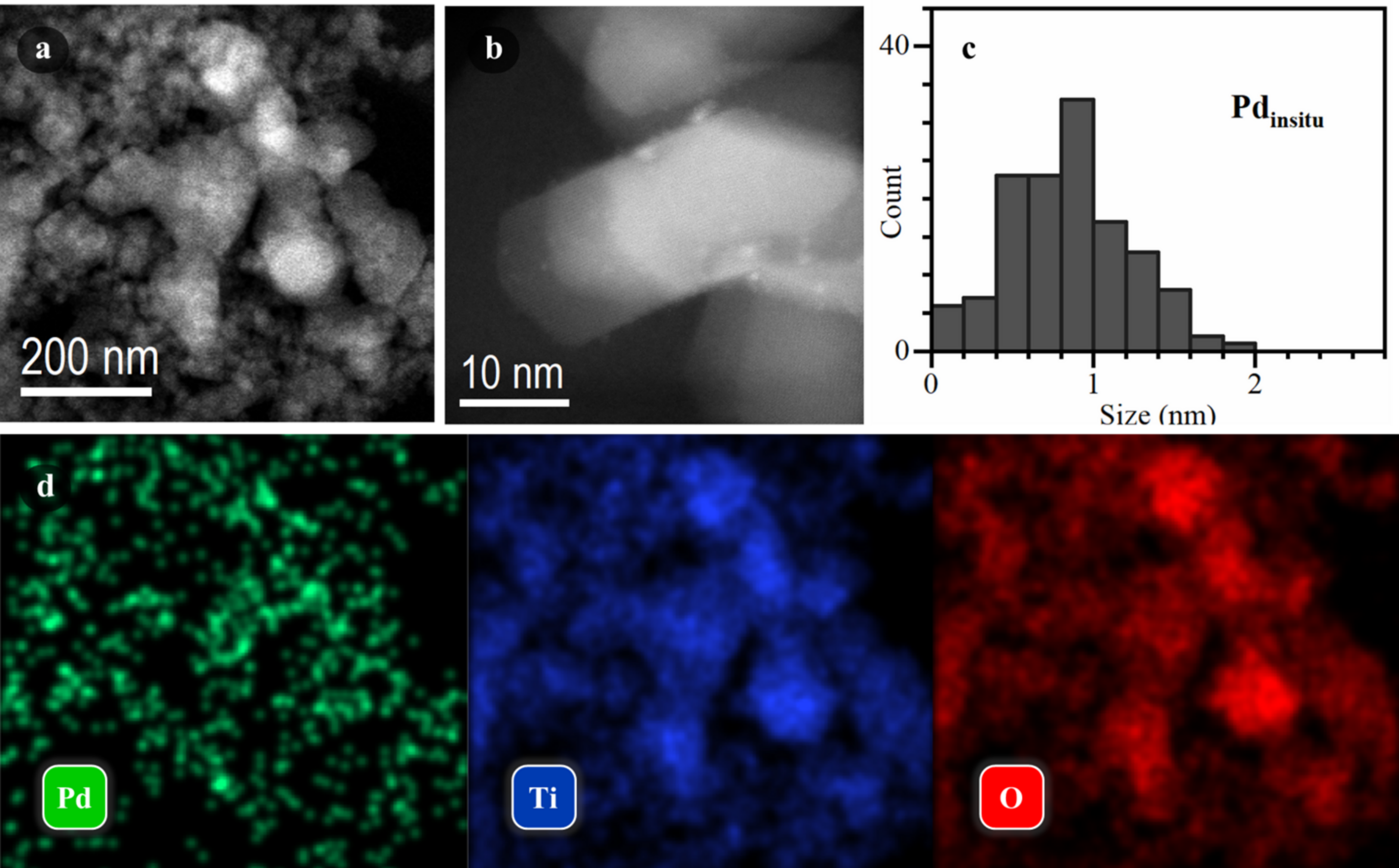
STEM characterization of the Pd_insitu_ sample. Low (*a*) and high (*b*) magnification HAADF-STEM images. (*c*) Histogram of Pd particle size distribution. (*d*) EDX elemental maps corresponding to the area shown in part (*a*).

**Figure 5:**
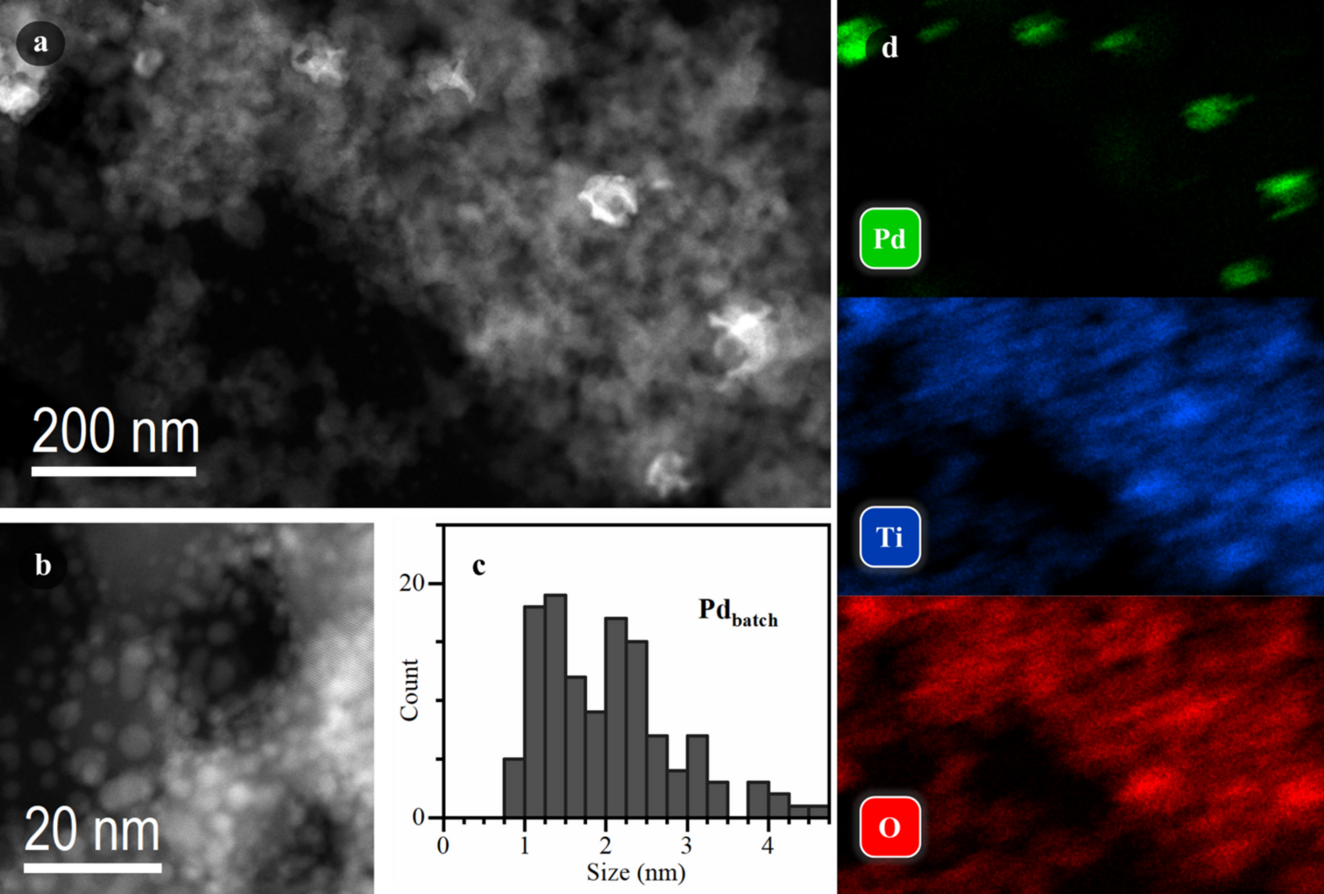
STEM characterization of the Pd_batch_ sample. Low (*a*) and high (*b*) magnification HAADF-STEM images. (*c*) Histogram of Pd particle size distribution. (*d*) EDX elemental maps corresponding to the area shown in part (*a*).

As the next step, we investigated the series of Pd/TiO_2_ samples using HAADF-STEM. The samples prepared *in situ* and collected from the window of the photocatalytic cell after XAS investigation will be referred to as Pd_insitu_/TiO_2_ and the sample synthesized in the conventional batch procedure as Pd_batch_/TiO_2_. As evident from Figs. 4 ▸ and 5 ▸ and additional micrographs shown in the supporting information, the *in situ* generated sample exhibited a homogeneous distribution of palladium over TiO_2_ with a narrow particle size distribution centered around 1 nm [Fig. 4 ▸(*c*)], in agreement with EXAFS data. In contrast, a very inhomogeneous distribution of Pd was observed for Pd_batch_/TiO_2_. This sample had bigger particles, which correlates with the EXAFS results, and a broader particle size distribution [Fig. 5 ▸(*c*)]. Moreover, particles or particle agglomerates with sizes up to 100 nm were detected, which were almost absent for Pd_insitu_/TiO_2_ (Fig. S8).

Further insights were gained from two additional samples that were generated *in situ* during XAS experiments following a similar procedure as for Pd_insitu_/TiO_2_ but using a lower irradiance of 80 and 200 W m^−2^. Unfortunately, a smaller degree of Pd incorporation into TiO_2_ limited the interpretation of XAS data (Fig. S9), although the presence of Pd(0) species could be unambiguously detected for the sample generated with 200 W m^−2^ irradiation, based on XANES spectra. HAADF-STEM data of these samples highlighted the presence of ultra-small Pd clusters (<1 nm) or single-atom Pd sites (Fig. S10), confirming the photodeposition mechanism proposed in the previous section. Bigger particles obtained for Pd_batch_/TiO_2_ can be explained by the fact that part of Pd was reduced in the liquid phase, acting as nucleation centers that facilitated the growth of larger particles that were then deposited on TiO_2_. This correlates with our recent study on Pt-based samples (Kozyr *et al.*, 2023 ▸), where photoinduced Pt reduction and formation of Pt nanoparticles were observed even in the absence of TiO_2_. Conversely, in Pd_insitu_/TiO_2_, the interaction of the solution with UV light was minimized since the radiation was mainly absorbed by the TiO_2_ layer. Thus, photoexcited TiO_2_ could act by providing the binding sites for Pd(0) single atoms while the maximum particle size could be limited due to the interaction with the support.

## 4. Conclusions

We have presented an efficient approach for *in situ* generation of Pd/TiO_2_ photocatalysts that allows all Pd sites to be equally accessible by both X-ray and UV radiation. *In situ* generation of the Pd/TiO_2_ sample using TiO_2_ immobilized on the front window of the photocatalytic cell was shown to produce a homogeneous distribution of Pd over the photoactive support, with a narrow particle size distribution and an average particle size of ∼1 nm. In contrast, bigger particles and a more inhomogeneous distribution were observed for batch synthesis with similar parameters. *In situ* XAS spectroscopy complemented by HAADF-STEM data revealed the formation of either single-atom Pd(0) sites or ultra-small Pd(0) clusters, as soon as Pd species were photodeposited on TiO_2_, that subsequently acted as nucleation sites for the growth of nanometre-sized Pd particles. The state of Pd in the presence of water and hole scavenger (formic acid) was identified as Pd hydride.

## Supplementary Material

Video abstract. DOI: 10.1107/S1600577524004788/ok5116sup1.mp4

Figures S1 to S10 and Table S1. DOI: 10.1107/S1600577524004788/ok5116sup2.pdf
