# Supplementary material for: In situ photodeposition of ultra-small palladium particles on TiO2
Source: J Synchrotron Radiat. 2024 Jul 15;31(Pt 5):1071–7. doi: 10.1107/S1600577524004788 (PMC11371036; doi:10.1107/S1600577524004788)
Supplement: Supplementary file 2 [file s-31-01071-sup2.pdf]

JOURNAL OF SYNCHROTRON RADIATION

Volume 31 (2024)

Supporting information for article:

In situ photodeposition of ultra-small palladium particles on TiO2

Elizaveta Kozyr, Sara Martí-Sánchez, Alina Skorynina, Jordi Arbiol, Carlos Escudero, Lorenzo Mino and Aram Bugaev

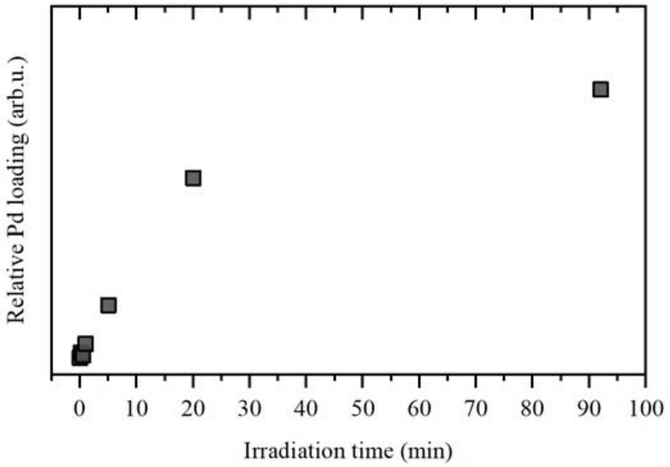

Figure S1 Relative changes in the Pd loading on TiO_2 as a function of irradiation time estimated based on the decrease of the Pd edge-jump in XAS signal due to inhomogeneities of the TiO_2 layer.

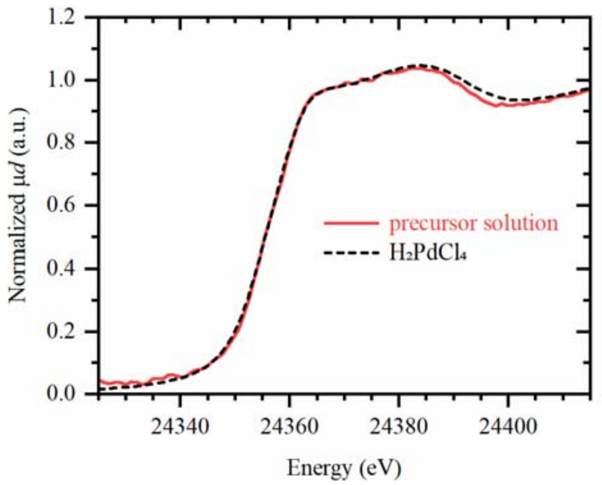

Figure S2 XANES spectra of the solution of palladium precursor, measured in transmission mode, compared with the H_2PdCl_4 reference.

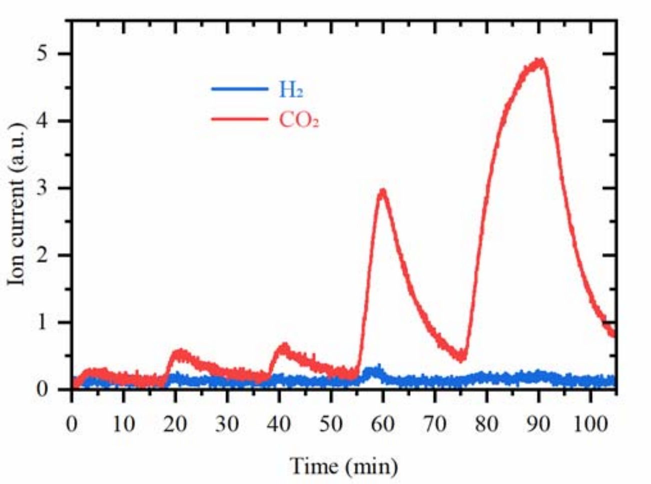

Figure S3 Mass spectrometry data for *m*/*Z* ratios of 2 (blue) and 44 (red) collected during *in situ* photodeposition of palladium (corresponding to Figure 2 of the main text).

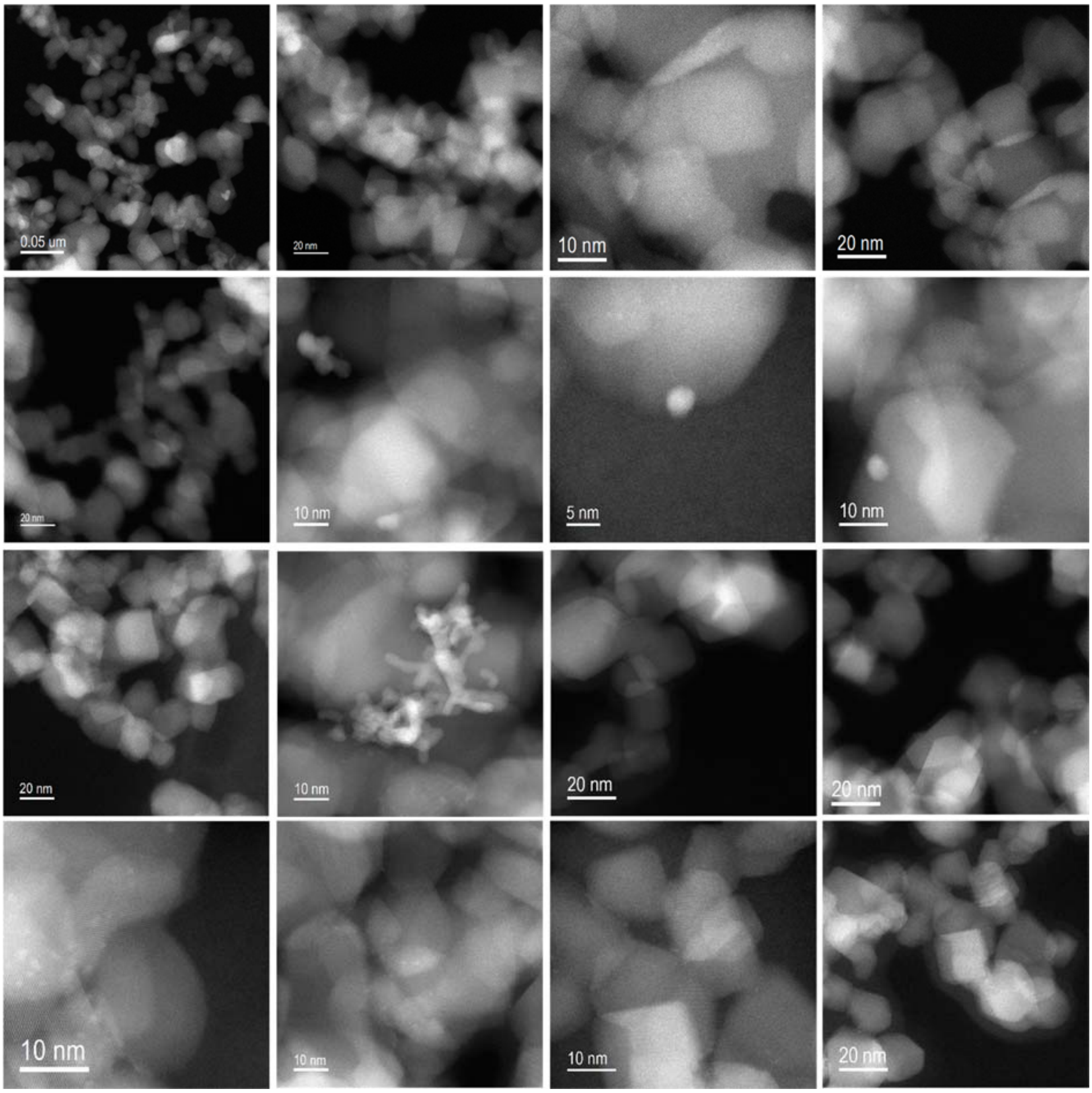

Figure S4 HAADF-STEM images of Pd_{insitu}/TiO_2 (UV irradiance 400 W/m^2).

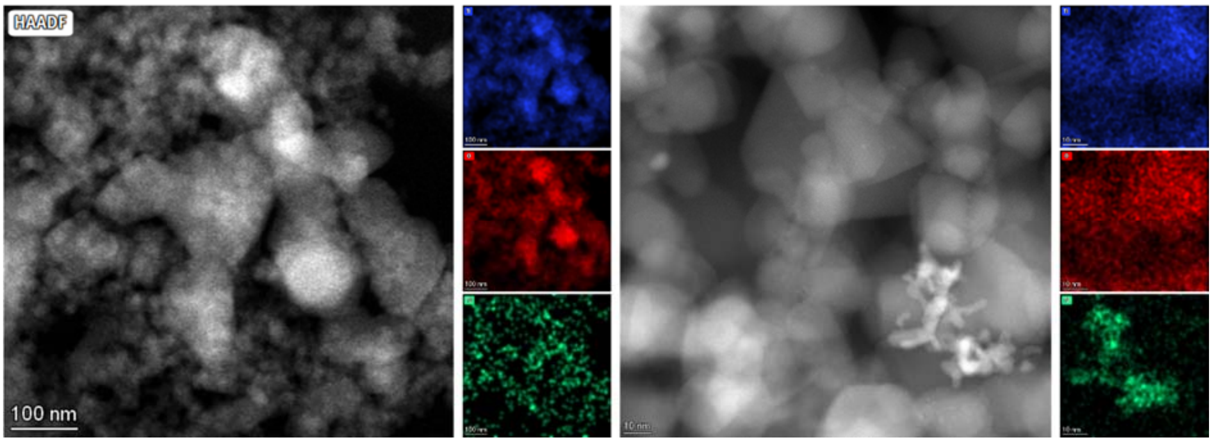

Figure S5 HAADF-STEM images and EDX maps (Ti – blue; O – red; Pd – green) for Pd_{insitu}/TiO_2.

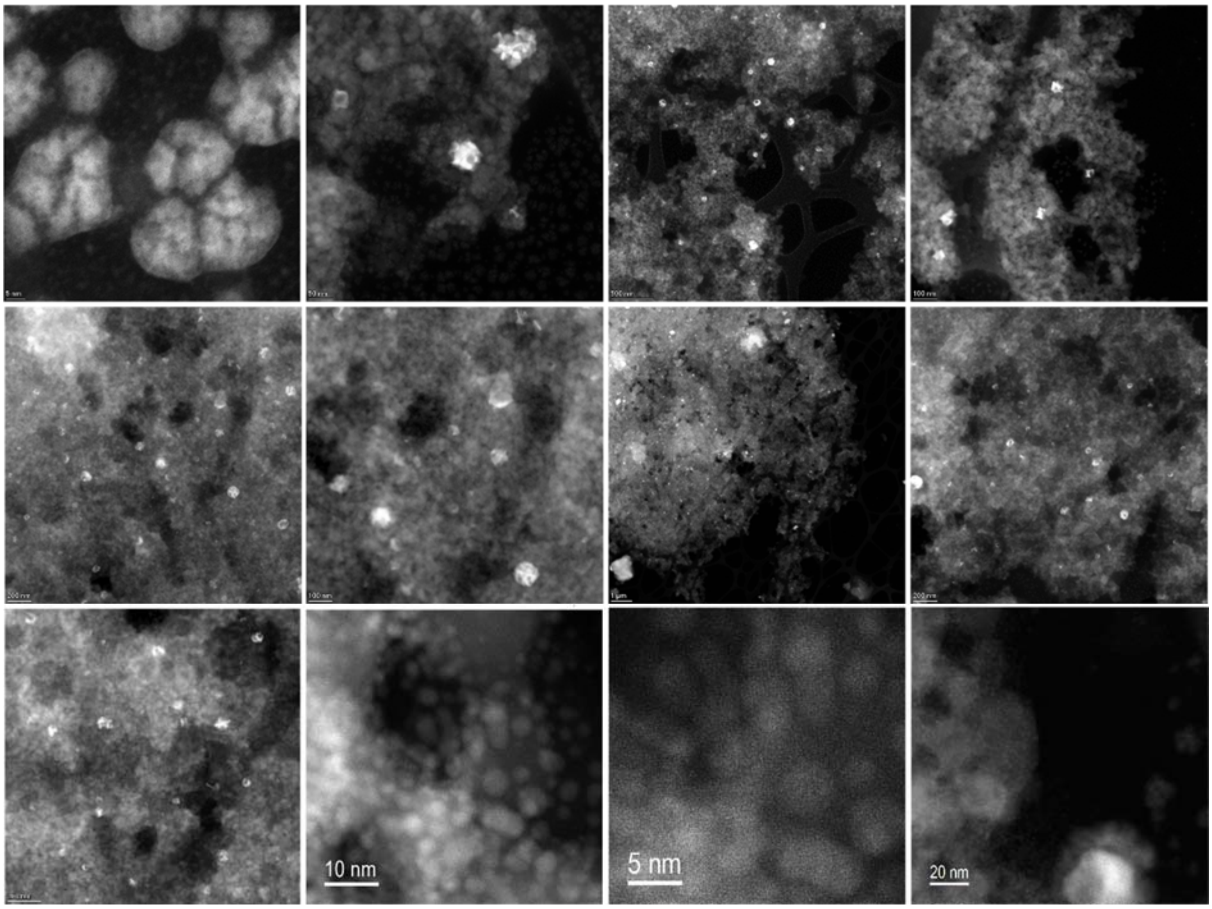

Figure S6 HAADF-STEM images of Pd_{batch}/TiO_2.

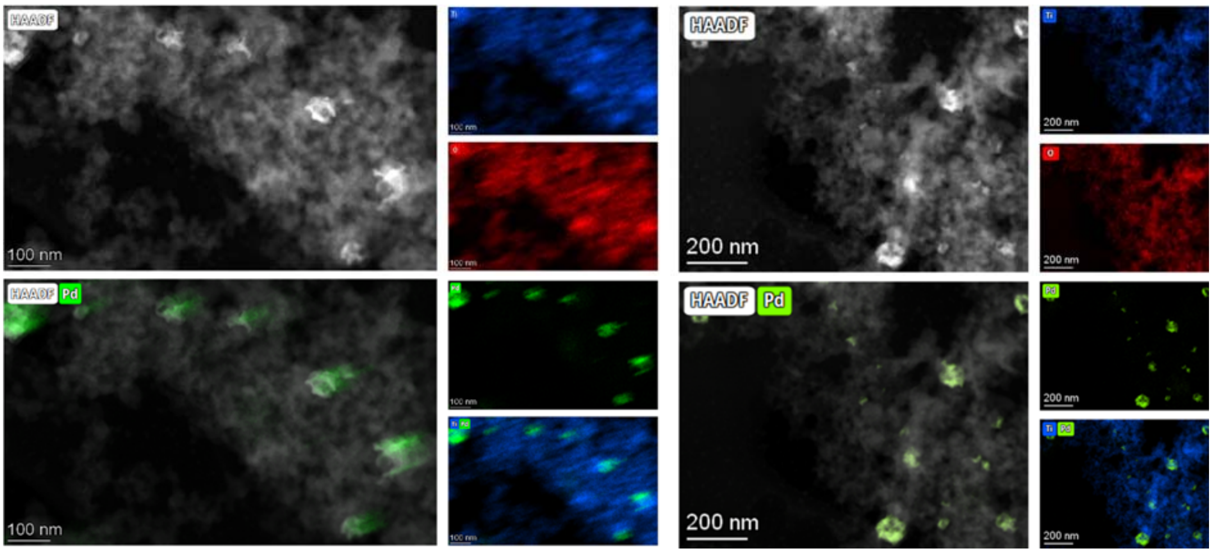

Figure S7 HAADF-STEM images and EDX maps (Ti – blue; O – red; Pd – green) for Pd_{batch}/TiO_2.

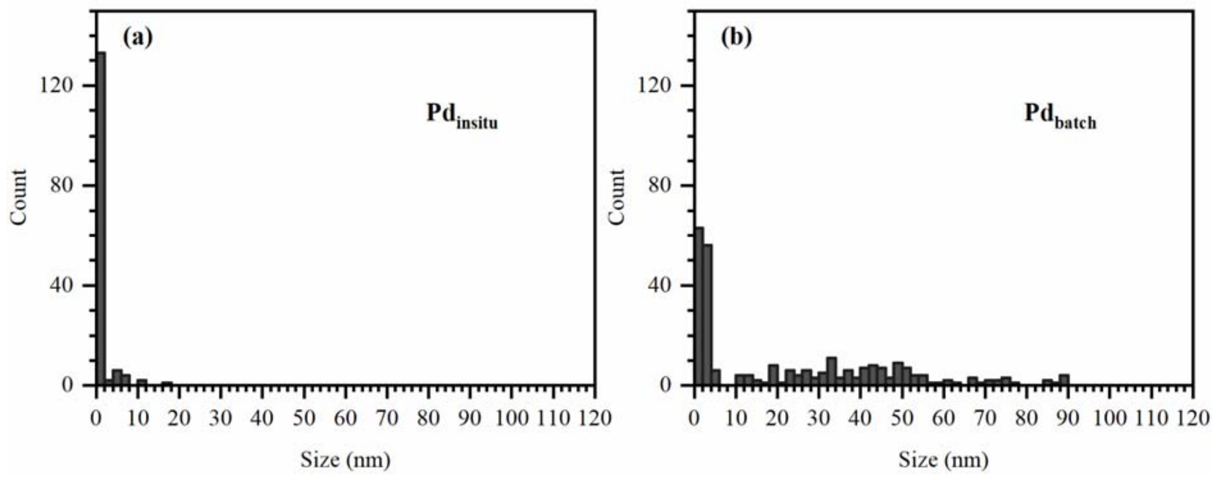

Figure S8 Histograms of particle size distribution for Pd_{insitu}/TiO_2 (a) and Pd_{batch}/TiO_2 (b) in a wider range with respect to Figure 6 of the main text.

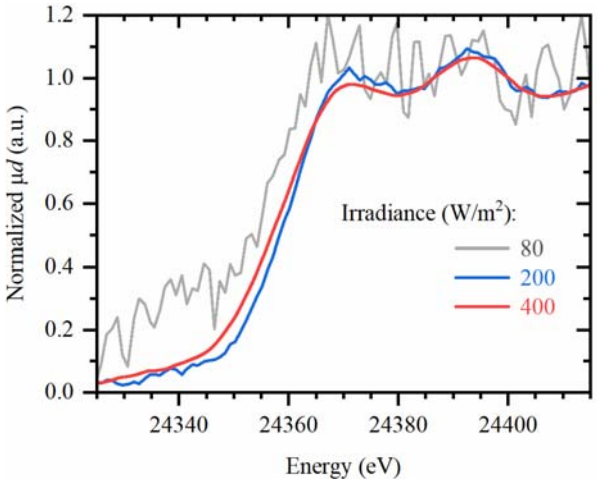

Figure S9 XANES spectra of *in situ* generated samples after 20 min of UV illumination with different irradiance (400, 200 and 80 W/m^2).

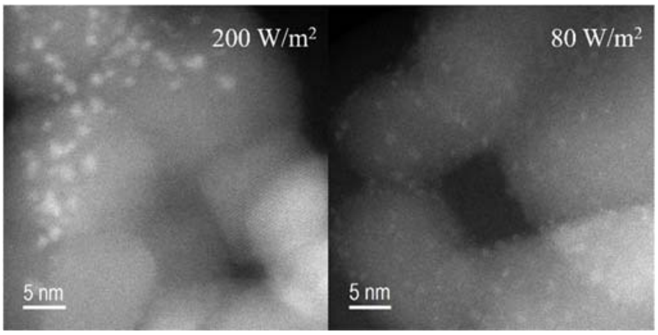

Figure S10 HAADF-STEM images *in situ* samples generated with UV 200 and 80 W/m^2 irradiance.

Table S1 Atomic and mass fractions estimated from EDX data for Pd_{insitu} sample.

Element	Series	Atomic fraction (%)	Mass fraction (%)
O	K	66.49 ± 3.76	39.8 ± 4.03
Ti	K	33.43 ± 3.77	59.86 ± 4.07
Pd	L	0.09 ± 0.02	0.34 ± 0.08
